# Integrated analysis of long non-coding RNA-microRNA-mRNA competing endogenous RNAregulatory networks in thromboangiitis obliterans

**DOI:** 10.1080/21655979.2021.2002497

**Published:** 2021-12-09

**Authors:** Bo Chen, Ying Deng, Bo Wang, Zhongyi Tian, Jindong Tong, Bo Yu, Weijun Shi, Jingdong Tang

**Affiliations:** a Department of Vascular Surgery, Shanghai Pudong Hospital, Fudan University Pudong Medical Center, Shanghai, China; b Shanghai Key Laboratory of Vascular Lesions Regulation and Remodeling, Shanghai Pudong Hospital, Fudan University Pudong Medical Center, Shanghai, China

**Keywords:** Thromboangiitis obliterans, lncRNA, miRNA, ceRNA network

## Abstract

Thromboangiitis obliterans (TAO) is a non-atherosclerotic, segmental, chronic vascular inflammatory disease. Our aim was to explore the underlying mechanisms of long non-coding RNA (lncRNA)-related competing endogenous RNAs (ceRNAs) in TAO. Six blood samples were collected from patients with TAO and healthy individuals (three for each category). Total RNA was extracted from the blood of each participant and sequenced. Differentially expressed lncRNAs (DE-lncRNAs) and miRNAs (DE-miRNAs) were screened, and ceRNA networks associated with TAO were constructed. Thereafter, the genes in the ceRNA network were subjected to functional analyses. Finally, a ceRNA relationship (lncRNA NEAT1-hsa-miR-1-3p-mRNA GNA12) was selected for further validation. Analysis revealed that 347 DE-lncRNAs (150 downregulated and 197 upregulated) and 16 DE-miRNAs (3 downregulated and 13 upregulated) were identified in TAO. Further, TAO-associated ceRNA networks, which included 219 lncRNAs, 6 miRNAs, and 53 mRNAs, were proposed and subjected to gene annotation and pathway analysis. Additionally, NEAT1 and GNA12 levels were significantly upregulated, while miR-1-3p levels were evidently downregulated in TAO patients, as compared with those in healthy controls. Dual luciferase reporter assays showed that NEAT1, miR-1-3p, and GNA12 interacted with each other. We report potential TAO-associated ceRNA regulatory networks and suggest activation of NEAT1/miR-1-3p/GNA12 signaling as a novel mechanism for TAO progression.

## Introduction

Thromboangiitis obliterans (TAO), also called Buerger’s disease, is a non-atherosclerotic, segmental, chronic vascular inflammatory disease that mainly affects the distal middle and small arteries and veins of the arms and legs [1]. TAO, characterized by ischemic pain, ulceration, or gangrenous plaques in the distal extremities, is a documented worldwide phenomenon with highest prevalence in the Middle and Far East [2]. Although the etiology and underlying mechanism of TAO remain unclear, previous studies have indicated that smoking is closely linked to the manifestation and progression of TAO [3]. Currently, the tools used to identify TAO are complex and involve intricate procedures, such as computed tomography (CT) angiography, magnetic resonance angiography, digital subtraction angiography, and segmental manometry [4]. These burdensome detection procedures are time-consuming and hinder the quick and efficient treatment of TAO. Additionally, one effective treatment for TAO is the cessation of smoking accompanied by drug treatment [5], such as vascular dilators (cilostazol), antiplatelet drugs (clopidogrel), and thrombolytic drugs (urokinase) [6]. However, no long-term benefit of these drugs has been confirmed [7], although they contribute to a number of side effects. Thus, it is imperative to identify the molecular mechanisms of TAO to facilitate the development of novel therapeutic targets and pathways for TAO treatment.

Recently, accruing evidence has indicated that non-coding RNAs, including long non-coding RNAs (lncRNAs) and microRNAs (miRNAs), play essential roles in various cellular processes and functions, including cell proliferation, migration, invasion, and apoptosis [8,9]. MiRNAs, a subset of small non-coding RNAs, bind with the 3′-untranslated region (3′-UTR) of their target mRNAs, thereby triggering translation inhibition or mRNA degradation [10]. LncRNAs, a type of non-coding RNA with a length of more than 200 nt, can serve as competing endogenous RNAs (ceRNAs) to regulate the interaction between miRNA and their target mRNAs by sponging miRNAs [11,12]. Previous studies have reported that an imbalance in a ceRNA network can influence cellular functions and may result in the onset and progression of diseases [13,14]. Yang et al. [15] have demonstrated that LINC01133 inhibits the development and metastasis of gastric carcinoma (GC) by acting as a ceRNA for miR-106a-3p to upregulate APC expression and the Wnt/β-catenin pathway and may be a potential biomarker for GC prognosis. Another report has described the construction of a ceRNA network related to hepatocellular carcinoma (HCC) prognosis by utilizing five different datasets associated with HCC and revealed that four lncRNAs (CRNDE, DLX6-AS1, MYCNOS, and LINC00221) and two mRNAs (SHCBP1 and CCNB1) can serve as prognostic biomarkers for HCC patients [16]. However, the roles and underlying mechanisms of lncRNA-related ceRNAs in TAO still remain unclear.

Therefore, in this study, we aimed to explore the underlying mechanisms of lncRNA-related ceRNAs in TAO. Total RNA extracted from the blood of TAO patients and healthy controls was sequenced, and lncRNA and miRNA profiles were screened for differentially expressed lncRNAs (DE-lncRNAs) and miRNAs (DE-miRNAs). Subsequently, ceRNA networks associated with TAO were constructed. Our results showed a relatively high differential expression of NEAT1, which has been reported to be closely related to the occurrence and development of diseases [17] such as cancer [18]. Based on the ceRNA network, hsa-miR-1-3p was selected for further analysis. Additionally, GNA12 has been reported to be involved in the phosphate pathway [19], which is associated with TAO progression [20]. Consequently, the ceRNA relationship involving lncRNA NEAT1-hsa-miR-1-3p-mRNA GNA12 was chosen for further authentication. Overall, the findings of this study can be helpful in improving our understanding of TAO occurrence and development in addition to providing potential targets and pathways for designing novel therapeutic strategies for TAO.

## Materials and methods

### Clinical specimens

From January 2019 to December 2019, TAO patients (n = 3) and healthy individuals (n = 3) were recruited from Shanghai Pudong Hospital, and 10 mL blood samples were collected from each. All patients with peripheral arterial disease that were clinically diagnosed with TAO according to Shionoya’s criteria were included [21]. The inclusion criteria were: onset of symptoms before 50 years of age, heavy smoking/smoking history, no other atherosclerotic risk factors, medium-size blood vessels with upper limb involvement, or superficial thrombophlebitis. Healthy blood donors with no history of thrombosis or arterial disease were randomly selected as the control group and were age-matched (<50 years of age, Table 1). We excluded the patients with peripheral arterial disease attributable to other causes, such as atherosclerosis, embolic vascular occlusion, dyslipidemia, vasculitis, trauma, radiation arteritis, or pro-coagulant status. The research protocol was approved by the Ethics Committee of Shanghai Pudong Hospital (No. WZ-003), and informed consent was obtained from all participants.

**Table 1.** The physiological and biochemical indexes of thromboangiitis obliterans (TAO) patients and healthy individuals

Types	Number	Sex	Age	complication	Therapeutic schedule	Drug usage	CPR (mg/dl)	SBP (mmHg)	DBP (mmHg)	blood glucose (mmol/L)	LDL (mmol/L)	TG (mmol/L)	ApoB (g/L)
TAO	1	Male	26	No	Lower extremity arterial balloon dilatation	Alprostadil	< 0.499	116	76	4.67	3.11	1.07	0.90
	2	Male	36	No	Lower extremity arterial balloon dilatation	Berprost sodium	0.55	138	90	4.68	2.62	0.84	0.76
	3	Male	36	No	Lower extremity arterial balloon dilatation	Alprostadil	9.92	131	91	4.42	2.22	1.61	0.81
healthy	1	Male	31	/	/	/	/	/	/	/	/	/	/
	2	Male	25	/	/	/	/	/	/	/	/	/	/
	3	Male	30	/	/	/	/	/	/	/	/	/	/

TAO: thromboangiitis obliterans; CRP: cardiopulmonary resuscitation; SBP: systolic blood pressure; DBP: diastolic blood pressure; LDL: low density lipoprotein; TG: triglyceride; ApoB: Apolipoprotein B.

### RNA isolation and sequencing

Total RNA was isolated from the blood of each subject using the RNAiso Plus kit (Takara Biomedical Technology Co., Ltd., Beijing, China) according to the manufacturer’s protocol. After that, the total RNA extracted from each blood sample was sent to Yanzai Biotechnology (Shanghai) Co. Ltd (Shanghai, China) for lncRNA and miRNA sequencing.

For miRNA library construction during sequencing, we used the TruSeq Small RNA Sample Prep Kit (Illumina). Then, the enrichment library was amplified by PCR, and sequencing connectors and index parts were added. The library was purified by gel electrophoresis. The Agilent High Sensitivity DNA Kit with Agilent 2100 Bioanalyzer was used to inspect the library, which was expected to only have a single peak without connectors. Thereafter, the library was quantified using the Quant-iT PicoGreen dsDNA Assay Kit, and the samples were sequenced on the Illumina platform. The raw data of sequencing were deposited in the NCBI SRA database, and the accession number is ‘PRJNA772535’.

### Screening of DE-lncRNAs and DE-miRNAs

After processing and analysis of lncRNA and miRNA sequencing data, differentially expressed lncRNAs (DE-lncRNAs) and differentially expressed miRNAs (DE-miRNAs) between healthy individuals and TAO patients were identified using the DESeq algorithm in the R package [22]. The thresholds to screen DE-lncRNAs were |log_2_Fold change (FC)| > 2 and *p* value < 0.05, and the criteria for DE-miRNA selection were |log_2_FC| > 1 and *p* value < 0.05. Next, these DE-lncRNAs and DE-miRNAs were subjected to Gene Ontology (GO) and Kyoto Encyclopedia of Genes and Genomes (KEGG) pathway analyses. Starbase (v3.0, http://starbase.sysu.edu.cn/) was used to screen the association pairs of lncRNA-miRNA. Subsequently, miRanda, an online tool, was used to predict the miRNA target mRNAs for the miRNAs screened out for further validation in the lncRNA-miRNA co-expression pairs, and the association pairs of mRNA-miRNA were obtained. After that, ceRNA regulatory networks were constructed using lncRNA-miRNA-mRNA co-expression pairs and visualized using Cytoscape (version 3.7.0). Finally, the ceRNA relationship of lncRNA NEAT1-miRNA hsa-miR-1-3p-mRNA GNA12 was chosen for further validation.

### Real-time quantitative PCR (RT-qPCR)

Total RNA (1 μg) was reverse transcribed into cDNA using the PrimeScript^TM^ II 1st Strand cDNA Synthesis Kit (Takara Biomedical Technology Co., Ltd., Beijing, China), according to the manufacturer’s instructions. The sequences of all primers are listed in Table 2.

**Table 2.** The sequences of all primers

Primer	Sequence (5ʹ-3ʹ)
hsa-miR-1-3p-RT	GTCGTATCCAGTGCAGGGTCCGAGGTATTCGCACTGGATACGACATACAT
hsa-miR-1-3p-F	TGGAATGTAAAGAAGT
hsa-miR-3124-5p-RT	GTCGTATCCAGTGCAGGGTCCGAGGTATTCGCACTGGATACGAC GACTTT
hsa-miR-3124-5p-F	TTCGCGGGCGAAGGC
hsa-miR-33a-3p-RT	GTCGTATCCAGTGCAGGGTCCGAGGTATTCGCACTGGATACGAC GTGATG
hsa-miR-33a-3p-F	GCGCAATGTTTCCACAGTG
hsa-miR-6786-3p-RT	GTCGTATCCAGTGCAGGGTCCGAGGTATTCGCACTGGATACGAC AGGCAG
hsa-miR-6786-3p-F	GCTGACGCCCCTTCTGATT
U6-hF	CTCGCTTCGGCAGCACA
U6-hR	AACGCTTCACGAATTTGCGT
LncRNA Neat-hF	TGGCTAGCTCAGGGCTTCAG
LncRNA Neat-hR	TCTCCTTGCCAAGCTTCCTTC
LncRNA RAD51-AS1-hF	TACTGCCGAAACAAACCACA
LncRNA RAD51-AS1-hR	CCACGACTCCCAAGAGGTAA
LncRNA SNHG22-hF	CGAAGGTCTCCTGTGAACCC
LncRNA SNHG22-hR	ACATGCTTTGGTGCTGCTTG
LncRNA MALAT1-hF	AAAGCAAGGTCTCCCCACAAG
LncRNA MALAT1-hR	GGTCTGTGCTAGATCAAAAGGCA
GNA12-hF	AGGGAACCAGTTTGCTCACC
GNA12-hR	GAGCCTACAGAGCCAAACCA
GAPDH-hF	TGACAACTTTGGTATCGTGGAAGG
GAPDH-hR	AGGCAGGGATGATGTTCTGGAGAG

For miRNA levels, the stem loop RT-PCR method was used, and U6 served as a reference gene. First, a 10 μL denaturing reaction solution was prepared and used for miRNA reverse transcription. The total volume for miRNA reverse transcription reaction was 20 μL, including 10 μL denaturing reaction solution, 4 μL of 5X PrimeScript II buffer, 0.5 μL RNase inhibitor (40 U/μL), 1 μL PrimeScript II RTase (200 U/μL), and 4.5 μL RNase free dH_2_O. The temperature protocol for miRNA reverse transcription was 42°C for 60 min, followed by 95°C for 5 min. Reverse transcriptase was used for qPCR, which was initiated at 95°C for 5 min, followed by 40 cycles of 95°C for 10 s and 60°C for 30 s.

For lncRNA and mRNA GNA12 expression, glyceraldehyde-3-phosphate dehydrogenase (*GAPDH*) was used as a housekeeping gene. The thermal cycling protocol used for lncRNA and GNA12 reverse transcription involved incubation of the reverse transcription mix at 37°C for 60 min and 85°C for 5s. RT-qPCR was initiated at 95°C for 3 min, followed by 40 cycles at 95°C for 10 s and 60°C for 30 s. The levels of NEAT1, MALAT1, RAD51-AS1, SNHG22, hsa-miR-1-3p, hsa-miR-6786-3p, hsa-3124-5p, hsa-miR-33a-3p, and GNA12 were calculated using the 2^−ΔΔCt^ method [23].

### Dual luciferase reporter gene assay

The sequences of the WT/MUT NEAT1 lncRNA inserts, hsa-miR-1-3p mimics/negative control (NC) mimics, and WT/MUT GNA12 3ʹ-UTRs were designed and synthesized by Yanzai Biotechnology Co. Ltd. (Shanghai, China). The psiCHECK2 vector and pGL3-basic vector were provided by Yanzai Biotechnology (Shanghai) Co. Ltd. and were used to construct the psiCHECK2-NEAT1 reporter plasmids (psiCHECK2-NEAT1 WT/MUT constructs) and the 3ʹ-UTR GNA12 reporter plasmids (pGL3-GNA12 WT/MUT constructs), respectively. 293T cells were purchased from the Cell Bank, Chinese Academy of Sciences (Shanghai, China) and were used for cell transfection in the dual luciferase reporter gene assay, due to their convenient transfection method and high transfection efficiency. Cell transfection was performed as described previously [24]. Briefly, 293T cells were seeded in a 96-well plate at a density of 1 × 10^4^ cells/well and cultured overnight. The following day, the culture medium was changed to serum-free medium, and then psiCHECK2 (0.4 μg) or psiCHECK2-NEAT1 WT/MUT (0.4 μg) and pGL3-basic vector (0.4 μg) or pGL3-GNA12 WT/MUT (0.4 μg) were co-transfected to 293T cells with hsa-miR-1-3p mimics (100 nM) or negative control (NC) mimics (100 nM) using Lipofectamine 3000 (Thermo Fisher Scientific, MA, USA) according to the manufacturer’s protocols. After 6 h of transfection, the medium was replaced with complete medium. After culturing for another 48 h, the cells were harvested, separated from culture medium using centrifugation, resuspended in 100 μL reporter lysis buffer, and then the mixture was centrifuged at 15,000 × *g* for 5 min. Luciferase activity of the supernatant was measured using a dual-luciferase reporter system (Promega Corporation, WI, USA) as per the manufacturer’s protocol.

### Statistical analysis

Data are presented as the mean ± standard deviation (SD). All statistical analyses were performed using GraphPad Prism 5 (GraphPad Software, CA, USA). Student’s t-test was used to compare two groups, whereas one-way analysis of variance (ANOVA) followed by Tukey’s test was used to compare more than two groups. Statistical significance was set at *p*< 0.05.

## Results

### Identification and functional analyses of DE-lncRNAs in TAO

**Figure 1.**
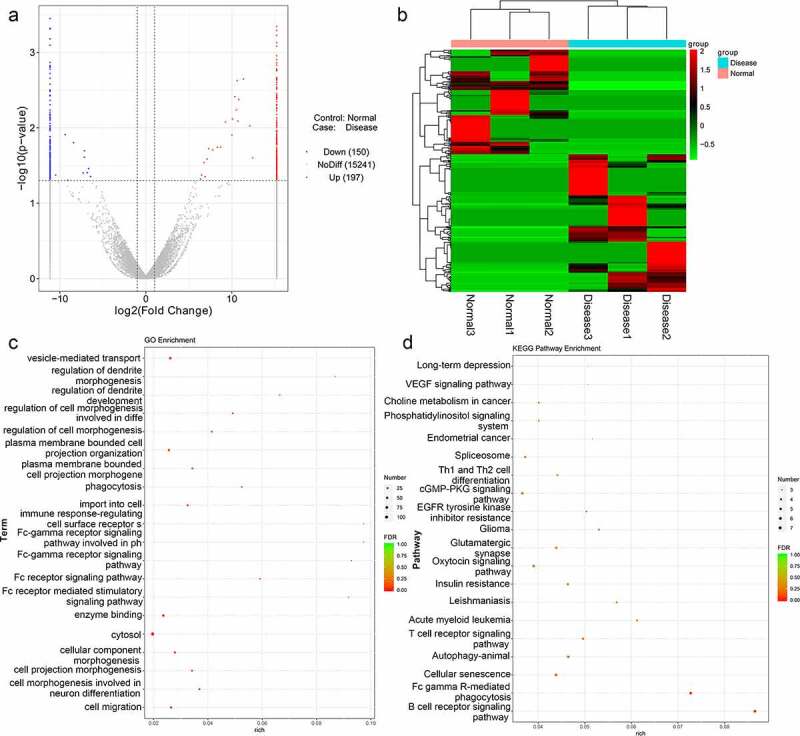
Analysis of thromboangiitis obliterans (TAO)-associated differentially expressed lncRNAs (DE-lncRNAs). (a) Volcano plot of DE-lncRNAs in TAO patients vs. healthy subjects. Blue and red represented the downregulated and upregulated genes, respectively. (b) The heatmap of DE-lncRNAs based on the expression level of DE-lncRNAs in TAO patients vs. healthy subjects. (c) Gene Ontology (GO) terms analysis of DE-lncRNAs. (d) Kyoto Encyclopedia of Genes and Genomes (KEGG) pathway enrichment of DE-lncRNAs

To identify differentially expressed lncRNAs of functional significance in TAO, RNA sequencing from total RNA extracted from TAO patients and healthy individuals was performed, and DE-lncRNAs associated with TAO were identified. The sample quality control data for the sequencing results are shown in Table 3. Our RNA sequencing data identified 347 DE-lncRNAs in TAO patients as compared with healthy individuals, with |log_2_FC| > 2 and *p* value < 0.05 as the thresholds; these included 150 downregulated lncRNAs (including AL355488.1 [ENST00000609909], SNHG22 [ENST00000615535], RAD51-AS1 [ENST00000533146]) and 197 upregulated lncRNAs (including NEAT1 [ENST00000501122], AC107068.1 [ENST00000563286], MALAT1 [ENST00000610481]) (Figure 1a). The heatmap distribution of these DE-lncRNA expressions is shown in Figure 1b. Subsequently, these DE-lncRNAs were subjected to GO and KEGG pathway enrichment analyses (Figure 1c, d). GO term analysis showed that these DE-lncRNAs were enriched in vesicle-mediated transport, regulation of dendrite morphogenesis, development, phagocytosis, Fc receptor signaling pathway, cytosol, cell morphogenesis involved in neuron differentiation, and cell migration, among various other phenomena (Figure 1c). The significantly enriched KEGG pathways of these DE-lncRNAs included long-term depression, VEGF signaling pathway, choline metabolism in cancer, phosphatidylinositol signaling system, autophagy-animal, cGMP-PKG signaling pathway, oxytocin signaling pathway, and B cell receptor signaling pathway (Figure 1d).

**Table 3.** The sample quality control data (QC data) of your sequenced data

Sample	Clean Reads No.	Clean Data (bp)	Clean Reads (%)
Normal 1	122,614,440	18,392,166,000	89.98
Normal 2	127,248,820	19,087,323,000	90.72
Normal 3	120,308,944	18,046,341,600	90.6
Disease 1	132,057,192	19,808,578,800	89.81
Disease 2	130,546,330	19,581,949,500	91.09
Disease 3	124,534,544	18,680,181,600	90.54

### Identification and functional analyses of TAO-associated DE-miRNAs

**Figure 2.**
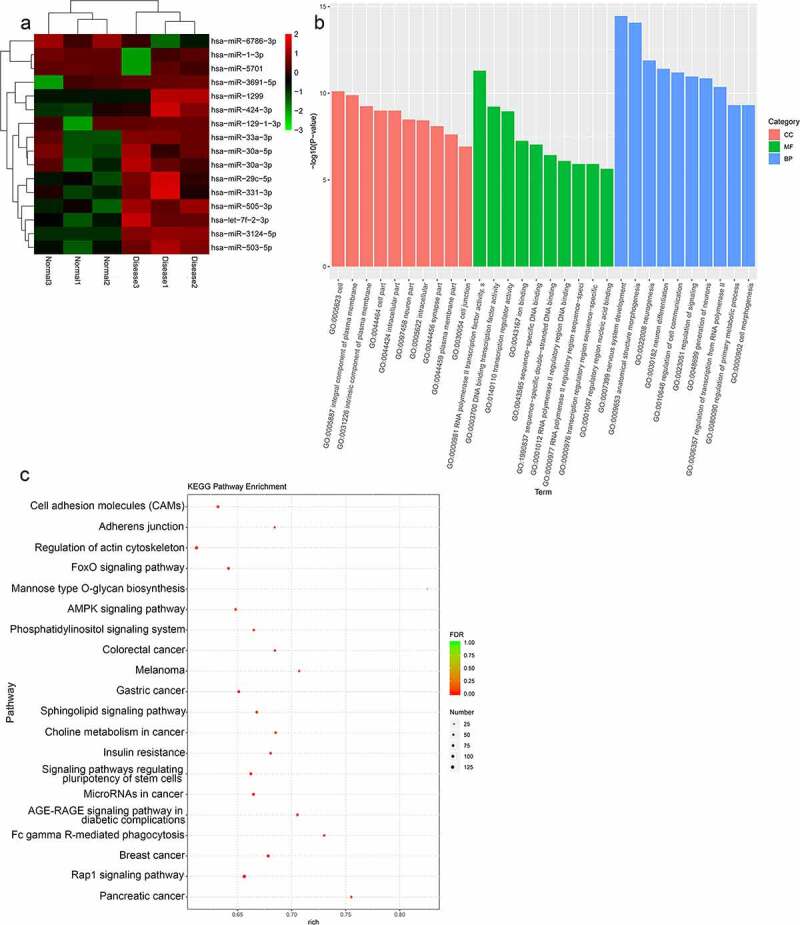
Analysis of TAO-associated differentially expressed miRNAs (DE-miRNAs). (a) The heatmap of DE-miRNAs, including 3 downregulated and 13 upregulated genes in TAO patients vs. healthy subjects. (b) GO terms analysis of DE-miRNAs. (c) KEGG pathway enrichment of DE-miRNAs

Further, to identify the differentially expressed miRNAs involved in the progression of TAO, the miRNA profiles were analyzed from the RNA sequencing data obtained from TAO patients and healthy controls. The analysis screened out 16 DE-miRNAs that were filtered based on the thresholds of |log_2_FC| > 1 and *p* value < 0.05 in TAO patients as compared with the healthy controls. These DE-miRNAs included three downregulated (hsa-miR-6786-3p, hsa-miR-1-3p, hsa-miR-5701) and 13 upregulated miRNAs (hsa-miR-3691-5p, hsa-miR-1299, hsa-miR-424-3p, hsa-miR-129-1-3p, hsa-miR-33a-3p, hsa-miR-30a-3p, hsa-miR-30a-5p, hsa-miR-29 c-5p, hsa-miR-331-3p, hsa-miR-505-3p, hsa-let-7 f-2-3p, hsa-miR-3124-5p, and hsa-miR-503-5p) (Figure 2a). GO-based functional annotation revealed that the target genes of these DE-miRNAs were located on ‘cell,’ ‘integral component of plasma membrane,’ and ‘neuron part’ for cell component (CC); ‘RNA polymerase II transcription factor activity,’ ‘DNA binding transcription factor activity,’ and ‘ion binding’ for molecular function (MF); and ‘nervous system development,’ ‘anatomical structure morphogenesis,’ ‘regulation of cell communication,’ and ‘generation of neurons’ for biological processes (BP) (Figure 2b). Furthermore, KEGG pathway analysis showed that these DE-miRNAs were enriched in ‘cell adhesion molecules (CAMs),’ ‘regulation of actin cytoskeleton,’ ‘FoxO signaling pathway,’ ‘AMPK signaling pathway,’ ‘phosphatidylinositol signaling system,’ ‘Rap1 signaling pathway,’ and ‘sphingolipid signaling pathway’ (Figure 2c).

### Analysis of ceRNA regulatory networks associated with TAO progression

**Figure 3.**
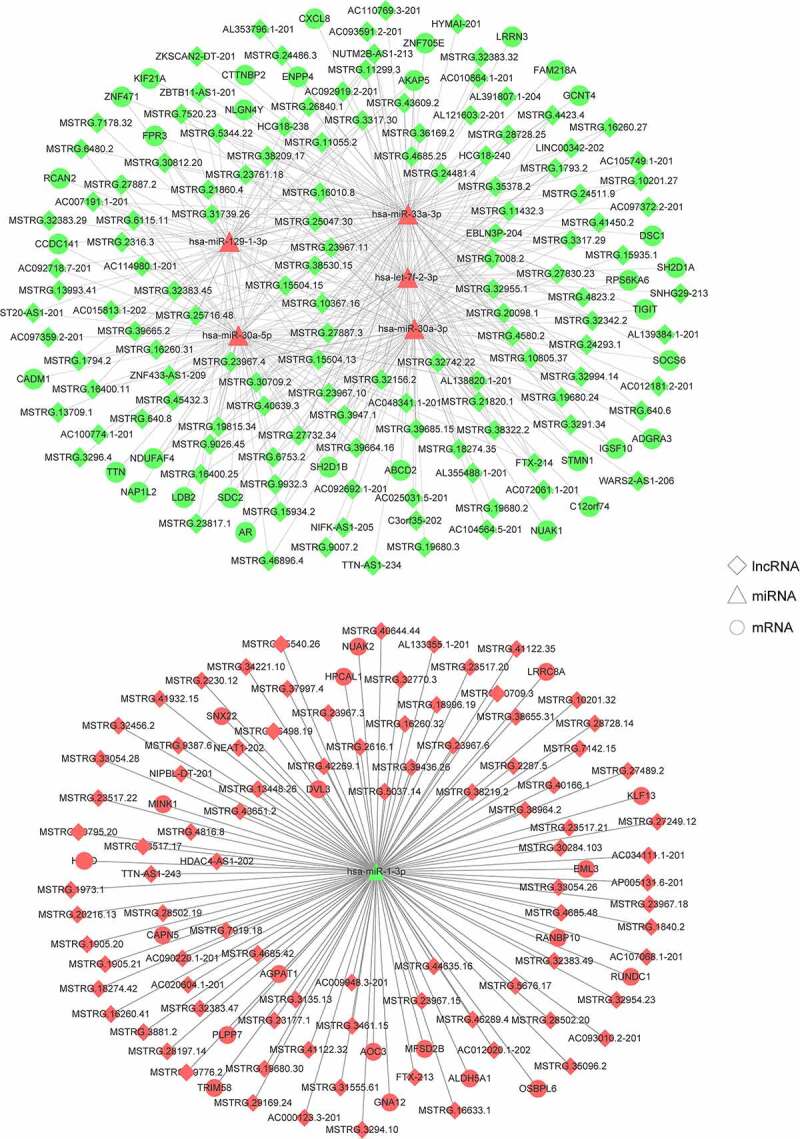
Construction of TAO-associated competitive endogenous RNA (ceRNA) regulatory networks. Rhombuses, triangles, and circles represent lncRNAs, miRNAs, and mRNAs, respectively. Red and green represent significant upregulation and downregulation in TAO patients vs. healthy subjects, respectively

**Figure 4.**
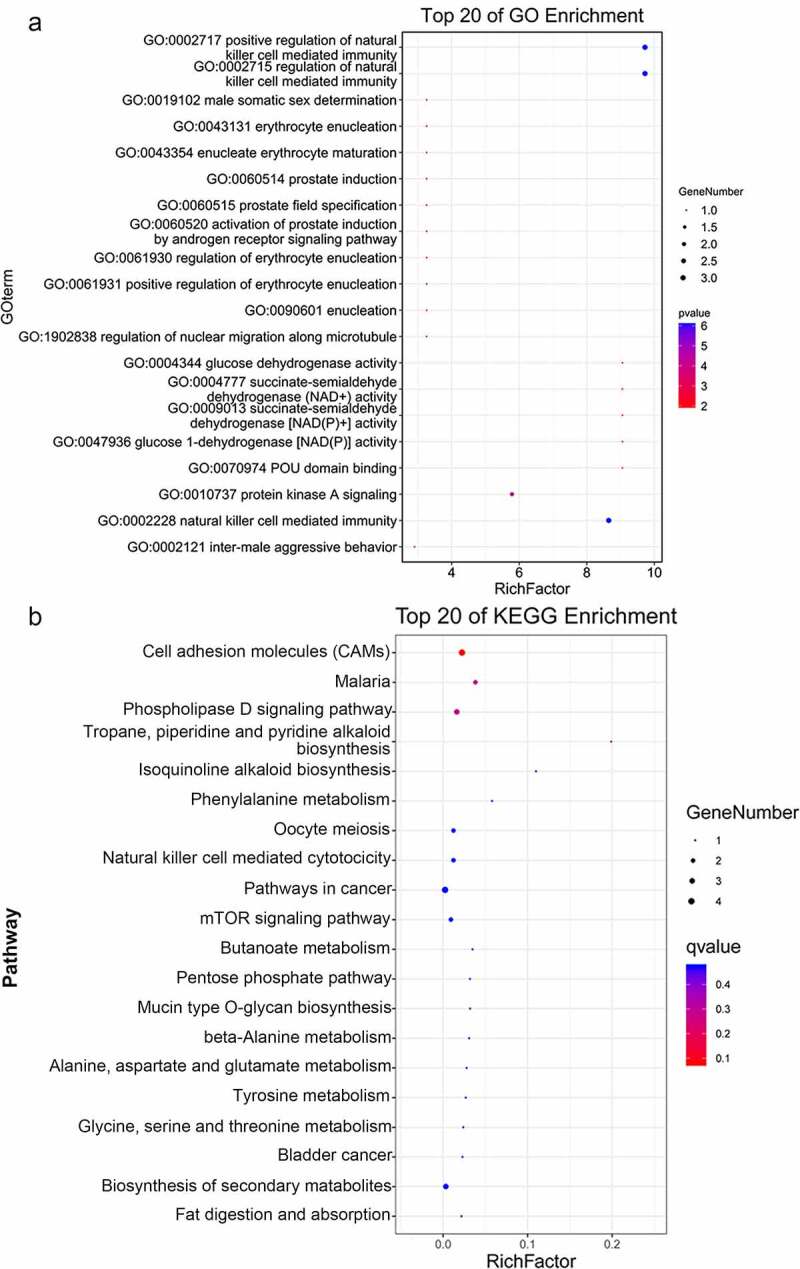
Functional analyses of the genes in the TAO-associated ceRNA networks. (a) GO terms analysis of the genes in the ceRNA networks involved in TAO progression. (b) KEGG pathway enrichment of the genes in the TAO-linked ceRNA networks

To establish ceRNA regulatory networks significantly associated with TAO, a comprehensive network analysis using the DE-lncRNA and DE-miRNA profiles obtained from sequencing total RNA samples from TAO patients and healthy controls was performed. The results of the network analysis revealed that 452 interaction pairs of lncRNAs-miRNAs, involving six DE-miRNAs and 219 lncRNAs, were associated with TAO progression upon retaining the profiles of DE-lncRNAs and DE-miRNAs with opposite expression directions. Subsequently, 77 interaction pairs of miRNA-mRNAs, containing 6 DE-miRNAs and 53 mRNAs, were screened by retaining the expression profiles of the DE-miRNAs and mRNAs in opposite directions. The ceRNA networks, which included 219 lncRNAs, 6 miRNAs, and 53 mRNAs, were then constructed (Figure 3), followed by performing functional analyses on the genes involved. GO annotations clearly suggested that these genes in the ceRNA networks were involved in the regulation of natural killer cell-mediated immunity and protein kinase A signaling, in addition to other GO-defined functions (Figure 4a). Additionally, KEGG pathway enrichment analysis indicated that these genes were also enriched in cell adhesion molecules (CAMs), phospholipase D signaling pathway, natural killer cell mediated cytotoxicity, mTOR signaling pathway, biosynthesis of secondary metabolites, and oocyte meiosis (Figure 4b).

### Validation of sequencing results and TAO-associated ceRNA relationship

**Figure 5.**
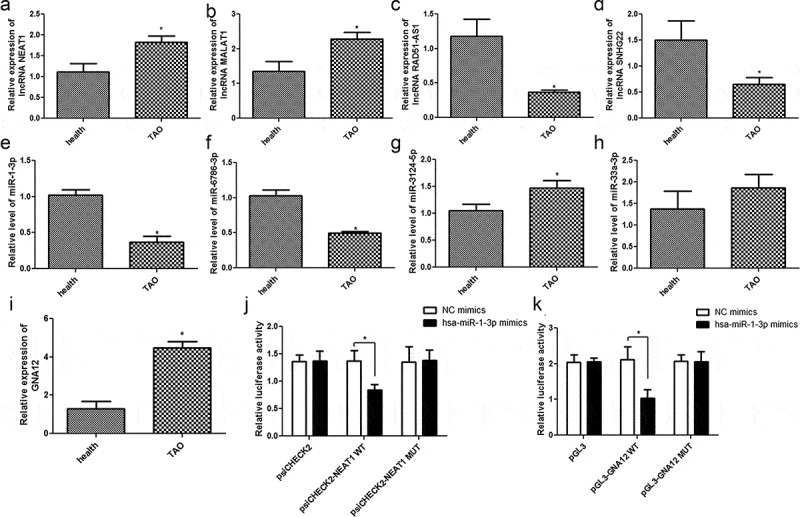
Validation of sequencing results and the ceRNA relationship of lncRNA NEAT1-hsa-miR-1-3p-mRNA GNA12 in TAO progression. The relative expressions of lncRNAs NEAT1 (a), MALAT1 (b), RAD51-AS1 (c), and SNHG22 (d) in healthy individuals and TAO patients. The relative levels of miR-1-3p (e), miR-6786-3p (f), miR-3124-5p (g), and miR-33a-3p (h) in healthy individuals and TAO patients. (i) The relative expression of GNA12 in healthy individuals and TAO patients. *p< 0.05 as compared with healthy individuals. (j) LncRNA NEAT1 interacts with miR-1-3p. *p< 0.05 as compared with negative control (NC) mimics. (k) GNA12 directly binds with miR-1-3p. *p< 0.05 as compared with NC mimics

In order to validate the results of our RNA sequencing data from TAO patients and healthy control RNA samples, two upregulated lncRNAs (NEAT1 and MALAT1) and miRNAs (miR-3124-5p and miR-33a-3p), as well as two downregulated lncRNAs (RAD51-AS1, SNHG22) and miRNAs (miR-1-3p, miR-6786-3p), were chosen for RT-qPCR verification. Our data clearly suggest that the expression of lncRNA NEAT1 and MALAT1 was significantly higher (*p*< 0.05, Figure 5a, b), while the expression of RAD51-AS1 and SNHG22 was significantly lower in TAO patients than in healthy individuals (*p*< 0.05, Figure 5c, d). Furthermore, the levels of miR-1-3p and miR-6786-3p in the TAO patients were evidently lower, and the level of miR-3124-5p was significantly higher (*p*< 0.05, Figure 5e-g) than that in healthy individuals. However, no significant difference was found in the levels of miR-33a-3p between the healthy individuals and TAO patients (*p*> 0.05, Figure 5h). Overall, these results indicated that the consistency in the RT-qPCR and sequencing-based expression analyses of lncRNA and miRNA were 100% and 75%, respectively, which showed the relatively high reliability of the sequencing results.

Furthermore, the ceRNA relationship of lncRNA NEAT1-miRNA hsa-miR-1-3p-mRNA GNA12 was selected for further validation. Our data showed a negative correlation between the expression of miR-1-3p and NEAT1 levels in TAO patients. In addition, the expression of GNA12 is significantly upregulated in TAO patients as compared to that in healthy individuals (*p*< 0.05, Figure 5i). Subsequently, the interactions among lncRNA NEAT1, hsa-miR-1-3p, and GNA12 were analyzed using a dual luciferase reporter gene assay. No significant difference was found between NC mimics and miR-1-3p mimics in the psiCHECK2 plasmid (*p* > 0.05). The results of the dual luciferase reporter gene assay showed that the relative luciferase activity in 293 T cells co-transfected with hsa-miR-1-3p mimics and psiCHECK2-NEAT1 WT plasmid was significantly lower than that in the NC mimic co-transfected cells (*p*< 0.05). However, after NEAT1 mutation, the relative luciferase activity in 293 T cells co-transfected with hsa-miR-1-3p mimics and psiCHECK2-NEAT1 MUT was restored to a similar level as that in the NC mimic co-transfected cells (*p*> 0.05, Figure 5j). Additionally, the relative luciferase activity in 293T cells co-transfected with the hsa-miR-1-3p mimics and pGL3-GNA12 WT plasmid was significantly lower than that in the NC mimic co-transfected cells. In contrast, co-transfection of 293 T cells with hsa-miR-1-3p mimics and pGL3-GNA12 MUT plasmid restored relative luciferase activity to levels similar to those in the NC mimic and pGL3-GNA12 WT plasmid co-transfected cells (*p*> 0.05, Figure 5k). These findings implied that lncRNA NEAT1, hsa-miR-1-3p, and mRNA GNA12 are involved in a potent lncRNA-miR-mRNA ceRNA regulatory network that might be of significant importance in the progression of TAO.

## Discussion

TAO is a type of thrombotic-occlusive and inflammatory peripheral vascular disease with unknown etiology that seriously affects public health and quality of life [25]. CeRNA networks represent important regulatory interactions among lncRNAs, miRNAs, and mRNAs [26]. They have been reported to be involved in the onset and development of many diseases [26,27]. However, the underlying ceRNA regulatory mechanisms in TAO still remain to be delineated. In this study, we have demonstrated the presence of ceRNA regulatory networks that are associated with TAO progression using next-generation RNA sequencing. We also discovered NEAT1/miR-1-3p/GNA12 as a novel pathway involved in the regulation of TAO progression.

Currently, ceRNA regulatory networks have facilitated the understanding of the underlying molecular mechanisms of various diseases and the identification of new biomarkers or therapeutic targets for them [28,29]. LncRNAs, considered an important class of ceRNAs, can serve as miRNA sponges to upregulate or downregulate the expression of protein-coding genes. In our study, transcriptome sequencing of RNA samples from TAO patients and healthy individuals identified 347 DE-lncRNAs (150 downregulated and 197 upregulated) and 16 DE-miRNAs (3 downregulated and 13 upregulated) associated with TAO progression. Subsequent network analyses proposed the existence of TAO-associated ceRNA regulatory networks, including 219 lncRNAs, 6 miRNAs (hsa-miR-1-3p, hsa-miR-129-1-3p, hsa-miR-33a-3p, hsa-miR-30a-5p, hsa-miR-30a-3p, and hsa-let-7 f-2-3p), and 53 mRNAs. Among the 6 miRNAs in the ceRNA regulatory networks, hsa-miR-1-3p and has-miR-129-1-3p have been reported to play important roles in tumor development, and hsa-miR-1-3p may be related to inflammation in atrial fibrillation [30]. A previous study showed that miR-33a-3p could serve as a novel circulating miRNA signature for the early detection of pancreatic neoplasia through RT-qPCR [31]. Another study found that miR-30a-3p and miR-30a-5p could serve as diagnostic biomarkers for heart failure and related diseases [32]. Moreover, Yao et al. [33] demonstrated via sequencing that hsa-let-7f-2-3p in circRNA-miRNA-mRNA networks was closely associated with synovial sarcoma pathogenesis. Taken together, we speculate that the 6 miRNAs, 219 lncRNAs, and 53 mRNAs in the ceRNA networks may be closely related to TAO occurrence and progression.

Additionally, GO and KEGG pathway analyses further revealed that that the genes in the TAO-associated ceRNA networks were related to the regulation of natural killer cell-mediated immunity, natural killer cell-mediated cytotoxicity, phospholipase D signaling, and mTOR signaling pathway. Natural killer cells are innate lymphocytes that are essential for host resistance to pathogens and cancer cells because of their ability to rapidly release inflammatory cytokines and kill infected or transformed cells [34]. A previous study has indicated that natural killer cell-mediated immune surveillance has a key inhibitory role in lymphoid malignancies [35]. Furthermore, Lee et al. [36] have shown that T cell-mediated immune inflammation is an important event in TAO progression. Phospholipase D is a regulator of intercellular signaling and metabolic pathways, especially in cells under stressful conditions, and phospholipid acid produced by phospholipase D plays multiple roles in many cellular functions such as vesicle transport, cell metabolism regulation, autophagy, and tumorigenesis [37]. A previous study has indicated that markers of oxidative stress, including serum ox-LDL, plasma and erythrocyte malondialdehyde, and plasma protein carbonyls, were significantly elevated in TAO patients as compared with those in healthy individuals [38], implying that oxidative stress may contribute to TAO development [25]. Additionally, mTOR, a serine/threonine kinase, regulates a variety of cellular functions and biological processes, such as cell proliferation, apoptosis, survival, growth, and autophagy [39]. Hadjadj et al. [40] have indicated that the mTOR signaling pathway participates in vascular remodeling and is associated with the pathogenesis of large-vessel vasculitis. These findings, combined with our results, suggest that regulation of natural killer cell-mediated immunity, natural killer cell-mediated cytotoxicity, phospholipase D signaling pathway, and mTOR signaling pathway may be closely related to the onset and progression of TAO.

Inflammation has a crucial stimulating effect on the pathogenesis of TAO [41,42], and previous studies have shown that ceRNA networks have regulatory roles in the progression of inflammation [43,44]. Zhang et al. [45] reported that lncRNA IGHCγ1 can act as a ceRNA, and its overexpression promotes macrophage inflammation by downregulating miR-6891-3p and upregulating TLR4, thus accelerating osteoarthritis pathogenesis. Another study has demonstrated that miR-223 can relieve the inflammatory response and thrombus in TAO rats by suppressing NLRP3 inflammasome expression [46]. Hence, ultimately, we chose a ceRNA network (lncRNA NEAT1-miRNA hsa-miR-1-3p-mRNA GNA12) associated with the development of TAO for further validation. We found that NEAT1 and GNA12 levels were significantly upregulated in TAO patients, while the levels of miR-1-3p in TAO patients were evidently lower than those in healthy controls. Furthermore, the dual luciferase reporter gene assay results confirmed that NEAT1, miR-1-3p, and GNA12 formed a ceRNA regulatory network by interacting with each other. NEAT1, a lncRNA transcribed from multiple endocrine tumor loci, has been reported to release chemokines and cytokines (IL-6, CXCL10) via the MAPK pathway [47] and has not been investigated in TAO. Zhang et al. [48] have shown that NEAT1 stimulates the activation of NLRP3, NLRC4, and AIM2 inflammasomes; enhances caspase-1 activation; and plays an important role in inflammation-related diseases such as TAO and other auto-inflammatory syndromes. GNA12, encoding a guanine nucleotide-binding protein, has been reported to be involved in the phosphate signaling pathway [19] and is upregulated in cancer cells and tumor tissues [49]. Additionally, miR-1 has been reported to promote endothelial inflammation and atherosclerosis formation [50]. Li et al. [51] have demonstrated that the lncRNA H19/miR-1-3p/CCL2 axis regulates the growth of lipopolysaccharide-induced normal human astrocytes, thereby participating in the inflammatory process of spinal cord injury. Taken together, our results suggest that activation of the NEAT1/miR-1-3p/GNA12 signaling pathway may play essential roles in the onset and progression of TAO. However, the mechanisms of the NEAT1/miR-1-3p/GNA12 signaling pathway in TAO need to be further explored *in vitro* and *in vivo*.

Further, we acknowledge that this study has some limitations. First, the sample size was small, and further experiments with a larger sample size are essential to substantiate our findings. The effects of the regulation of natural killer cell-mediated immunity, natural killer cell-mediated cytotoxicity, phospholipase D signaling pathway, and mTOR signaling pathway in TAO should be further explored. Additionally, the specific mechanisms of the NEAT1/miR-1-3p/GNA12 signaling pathway in TAO need to be investigated using *in vitro* and *in vivo* assays, and other lncRNA-related ceRNA networks in TAO should also be studied.

## Conclusion

Through analyzing ceRNA regulatory networks associated with TAO, we found that activation or suppression of the regulation of natural killer cell-mediated immunity, natural killer cell-mediated cytotoxicity, phospholipase D signaling pathway, and mTOR signaling pathway may play important roles in TAO progression. Additionally, NEAT1 and GNA12 levels were upregulated, and miR-1-3p levels were downregulated in TAO patients, compared to those in healthy controls, confirming their close association with TAO progression. We strongly believe that our findings will help to expand our understanding of the ceRNA regulatory mechanisms related to TAO and provide a basis for NEAT1/miR-1-3p/GNA12 as a novel therapeutic target pathway for the development of effective TAO therapy.

## Data Availability

The raw data of sequencing openly available in a public repository that issues datasets with the accession number of PRJNA772535 in the NCBI SRA database. Additionally, other data used and/or analyzed during the current study are available from the corresponding author on reasonable request.

## Highlights

- Phospholipase D signaling pathway and mTOR signaling pathway may play important roles in TAO progression.
- NEAT1/miR-1-3p/GNA12 may be a novel therapeutic target pathway for the development of effective TAO therapy.
- Our findings will help to expand understanding of the ceRNA regulatory mechanisms related to TAO.
